# Dermatopontin Influences the Development of Obesity-Associated Colon Cancer by Changes in the Expression of Extracellular Matrix Proteins

**DOI:** 10.3390/ijms23169222

**Published:** 2022-08-17

**Authors:** Victoria Catalán, Paula Domench, Javier Gómez-Ambrosi, Beatriz Ramírez, Sara Becerril, Amaia Mentxaka, Amaia Rodríguez, Víctor Valentí, Rafael Moncada, Jorge Baixauli, Camilo Silva, Javier Escalada, Gema Frühbeck

**Affiliations:** 1 Metabolic Research Laboratory, Clínica Universidad de Navarra, 31008 Pamplona, Spain; 2 CIBEROBN, Instituto de Salud Carlos III, 31008 Pamplona, Spain; 3 Obesity and Adipobiology Group, IdiSNA, 31008 Pamplona, Spain; 4 Department of Surgery, Clínica Universidad de Navarra, 31008 Pamplona, Spain; 5 Department of Anesthesia, Clínica Universidad de Navarra, 31008 Pamplona, Spain; 6 Department of Endocrinology & Nutrition, Clínica Universidad de Navarra, 31008 Pamplona, Spain

**Keywords:** obesity, colon cancer, dermatopontin, extracellular matrix remodelling, inflammation

## Abstract

Dysfunctional adipose tissue (AT) in the context of obesity leads to chronic inflammation together with an altered extracellular matrix (ECM) remodelling, favouring cancer development and progression. Recently, the influence of dermatopontin (DPT) in AT remodelling and inflammation has been proposed. We aimed to evaluate the role of DPT in the development of obesity-associated colon cancer (CC). Samples obtained from 73 subjects [26 lean (LN) and 47 with obesity (OB)] were used in a case-control study. Enrolled subjects were further subclassified according to the established diagnostic protocol for CC (42 without CC and 31 with CC). In vitro studies in the adenocarcinoma HT-29 cell line were performed to analyse the impact of pro- and anti-inflammatory mediators on the transcript levels of DPT as well as the effect of DPT on ECM remodelling and inflammation. Although obesity increased (*p* < 0.05) the circulating levels of DPT, its concentrations were significantly decreased (*p* < 0.05) in patients with CC. Gene expression levels of DPT in the colon from patients with CC were downregulated and, oppositely, a tendency towards increased mRNA levels in visceral AT was found. We further showed that DPT expression levels in HT-29 cells were enhanced (*p* < 0.05) by inflammatory factors (LPS, TNF-α and TGF-β), whereas the anti-inflammatory IL-4 decreased (*p* < 0.05) its expression levels. We also demonstrated that DPT upregulated (*p* < 0.05) the mRNA of key molecules involved in ECM remodelling (*COL1A1*, *COL5A3*, *TNC* and *VEGFA*) whereas decorin (*DCN*) expression was downregulated (*p* < 0.05) in HT-29 cells. Finally, we revealed that the adipocyte-conditioned medium obtained from volunteers with OB enhanced (*p* < 0.01) the expression of DPT in HT-29 and Caco-2 cells. The decreased circulating and expression levels of DPT in the colon together with the tendency towards increased levels in visceral AT in patients with CC and its influence on the expression of ECM proteins suggest a possible role of DPT in the OB-associated CC.

## 1. Introduction

Colon cancer (CC) represents the third most commonly diagnosed malignancy after breast and lung cancer and the second principal cause of cancer mortality [1]. Different risk factors associated with the development and prognosis of CC are preventable, comprising an excessive intake of red and processed meat, consumption of high-fat diets, a low intake of vegetables and fruits, physical inactivity, alcohol drinking, smoking and obesity [2,3,4]. Epidemiological studies evidenced a significant impact of obesity not only on the risk of developing gastrointestinal cancer but also on its diagnosis and treatment [5]. Specifically, dysfunctional adipose tissue (AT) in obesity has been closely associated with tumour growth and metastasis, with chronic and unresolved inflammation together with the altered extracellular matrix (ECM) remodelling and fibrosis constituting key factors in this association [6,7]. The ECM is a dynamic and tissue-specific structure that provides a scaffold to the cellular constituents and orchestrates multiple biochemical and biomechanical processes involved not only in tissue differentiation and homeostasis but also in disease progression [8,9,10,11]. The fibrotic ECM of AT in obesity is characterized by reduced plasticity due to the deposition of stiff ECM components by diverse cells including adipocytes, myofibroblasts, macrophages and the recently described fibro-inflammatory progenitors cells [12,13]. This dense and solid ECM structure exerts dysregulated biomechanical forces in the microenvironment promoting cancer cell migration and blood vessels deformation, optimal for tumour development and progression. In addition, the ECM in obesity constitutes a reservoir for different signalling molecules involved in cancer development [14].

Dermatopontin (DPT) is a tyrosine-rich and small molecular weight protein that comprises a considerable proportion of the non-collagenous ECM components [15]. We previously described a novel role for DPT in obesity influencing AT remodelling and inflammation [16]. In this line, a reduction in the accumulation of collagen in the ECM in the skin of *Dpt*-knockout mice was previously described [17] and *Dpt*-deficient mice with induced hepatic fibrosis exhibited less collagen deposition compared to wild-type animals [18]. A great variety of biological functions in both, physiological and pathological processes have been attributed to DPT due to its interaction with the transforming growth factor-β (TGF-β) and decorin together with its binding to integrin α3β1 and syndecan [19,20,21]. In particular, the effect of DPT on the increase of the activity of TGF-β in colon carcinogenesis suggests its tumour suppressor role and its function as a pre-receptor component of the TGF-β signalling pathway [22]. In this line, reduced expression of *DPT* in different neoplasms including hepatocellular carcinoma, ovarian, oral or breast cancer has been described [23,24,25,26].

To our knowledge, the function of DPT in obesity-associated CC has not yet been fully disentangled. We hypothesize that dysregulated levels of DPT in obesity may be associated with CC development. Thus, we aimed to examine whether obesity influences the serum levels of DPT and its mRNA levels in patients with CC. We also analysed the effect of pro- and anti-inflammatory factors on the gene expression levels of DPT and the impact of DPT on inflammation and ECM remodelling in HT-29 colon cancer cells. Finally, we explore the effect of the crosstalk between adipocytes and CC cells in relation to the expression of DPT.

## 2. Results

### 2.1. Colon Cancer Decreases DPT Circulating Levels and Its mRNA Expression in the Colon

Baseline characteristics of the study sample are shown in Table 1. Body weight, body mass index (BMI), estimated body fat and waist circumference were higher (*p* < 0.001) in individuals with obesity (OB) compared with lean (LN) volunteers. No differences in anthropometric and adiposity markers were found between patients with or without CC. Patients with OB and CC exhibited higher (*p* < 0.001) C-reactive protein (CRP) levels in comparison with LN and OB patients without CC. Concentrations of fibrinogen were increased (*p* < 0.01) in patients with OB with CC compared with volunteers with OB without CC. As expected, a significant increase in carcinoembryonic antigen (CEA) levels was found among patients with CC compared to controls.

**Figure 1:**
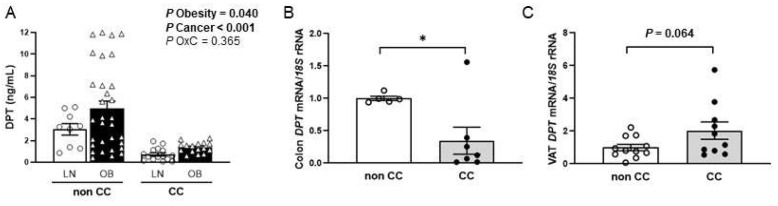
(**A**) Circulating levels of dermatopontin (DPT) of lean (LN) volunteers and patients with obesity (OB) classified according to the presence or not of colon cancer (CC) [LN-nonCC: n = 9; OB-nonCC: n = 31; LN-CC: n = 15; OB-CC: n = 16]. DPT gene expression levels in (**B**) colon [nonCC: n = 5; CC: n = 7] and (**C**) visceral adipose tissue (VAT) [nonCC: n = 11; CC: n = 10] in patients with and without CC. Differences between groups were analysed by two-way ANCOVA and by two-tailed unpaired Student’s *t*-tests. Bars represent the mean ± SEM. * *p* < 0.05.

Circulating DPT levels were increased (*p* = 0.040) in patients with OB and decreased (*p <* 0.001) in patients with CC (Figure 1A). No sexual dimorphism was found in serum concentrations of DPT (*p* = 0.359). A significant decrease in the expression of DPT was found in the colon of patients with CC compared to controls (*p* < 0.05) (Figure 1B). Since visceral AT (VAT) exhibits a fundamental role in obesity-associated inflammation and colon carcinogenesis, the gene expression of DPT in this AT depot was also studied. A tendency towards higher mRNA DPT levels was found in patients with CC, although differences were not statistically significant (*p* = 0.064) **(**Figure 1C).

### 2.2. Influence of Inflammation-Related Factors in the Expression of DPT in HT-29 Cells

**Figure 2:**
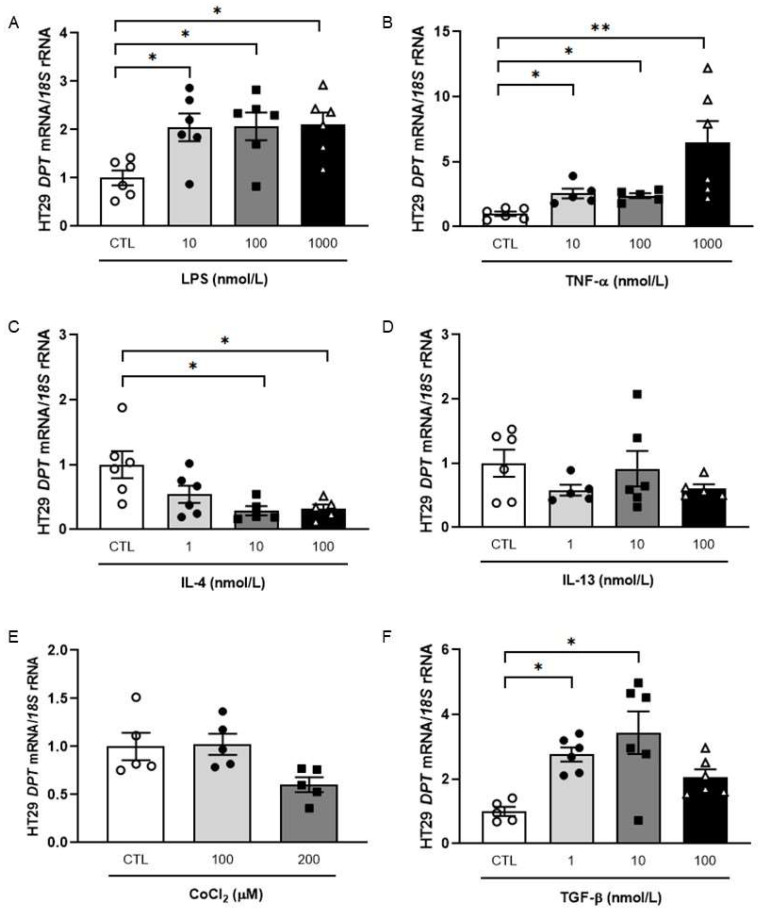
Effect of LPS (**A**), TNF-α (**B**), IL-4 (**C**), IL-13 (**D**), CoCl_2_ (**E**) and TGF-β (**F**) on mRNA levels of DPT in HT-29 colon cancer cells. Bar graphs are the mean ± SEM (n = 5–6 per group). One-way ANOVA followed by Dunnett’s *post hoc* tests was used to analyse differences between groups. * *p* < 0.05 and ** *p* < 0.01.

Inflammation constitutes a crucial hallmark of CC local progression enhancement, and thus, we evaluated whether pro- and anti-inflammatory factors that are altered in OB have an effect on DPT expression in HT-29 cells. The treatment of HT-29 cells with lipopolysaccharide (LPS) (*p* < 0.05) and with tumour necrosis factor (TNF)-α (*p* < 0.01) resulted in increased DPT mRNA levels (Figure 2A,B) whereas a downregulation after the treatment with the anti-inflammatory interleukin (IL)-4 (*p* < 0.05) was detected (Figure 2C). No significant differences in DPT mRNA expression after stimulation with IL-13 or under hypoxia were detected (Figure 2D,E). Importantly, a significant increase in DPT was found after the treatment with TGF-β at lower concentrations (Figure 2F).

### 2.3. Impact of DPT on the Expression of Key ECM Remodelling-Related Genes in HT-29 Cells

**Figure 3:**
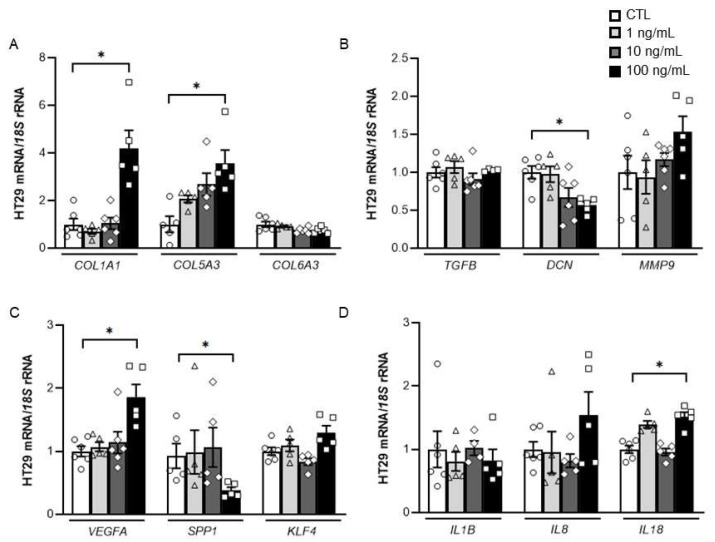
Effect of DPT treatment on the expression levels of (**A**) Collagen (*COL*)-*1A1*, *COL5A3* and *COL6A3*; (**B**) transforming growth factor-β (*TGFB*), decorin (*DCN*) and matrix metalloproteinase (*MMP*)-*9*; (**C**) vascular endothelial growth factor A (*VEGFA*), osteopontin (*SPP1*) and Kruppel-like factor 4 (*KLF4*) and (**D**) interleukin (*IL)-1B*, *IL8* and *IL18* in colon cancer cells. Values are the mean ± SEM (n = 5–6 per group). Differences between groups were analysed by one-way ANOVA followed by Dunnett’s *post hoc* tests. * *p* < 0.05.

Tumour cells were treated with increasing concentrations of DPT to explore whether DPT itself influences the expression of molecules closely related to the remodelling of the ECM and the inflammatory response. We found that the treatment with DPT increased (*p* < 0.05) the mRNA levels of the ECM remodelling-related genes collagen type 1 α1 chain (*COL1A1*), collagen type 5 α3 chain (*COL5A3*) and vascular endothelial growth factor A (*VEGFA*) together with a significant decrease of decorin (*DCN*) (*p* < 0.01) and osteopontin (*SPP1*) (*p* < 0.05). No effect of DPT on the analysed inflammation-related genes was observed except for an increase in the expression levels of interleukin (*IL*)-*18* (Figure 3).

Remarkably, gene expression levels of *COL1A1*, *COL5A3* and *TGFB* were upregulated (*p* < 0.05) after the treatment with DPT in Caco-2 cells, another common cell line used to reproduce the features of the tumoural bowel epithelium. The expression of the inflammatory markers IL1B (*p* < 0.01) and IL8 (*p* < 0.05) were increased in DPT treated-Caco-2 cells. No effect of DPT on the rest of analyzed genes were found (Supplemental Figure S1).

### 2.4. Adipocyte-Conditioned Media Upregulate DPT Expression Levels in Tumour Cells

**Figure 4:**
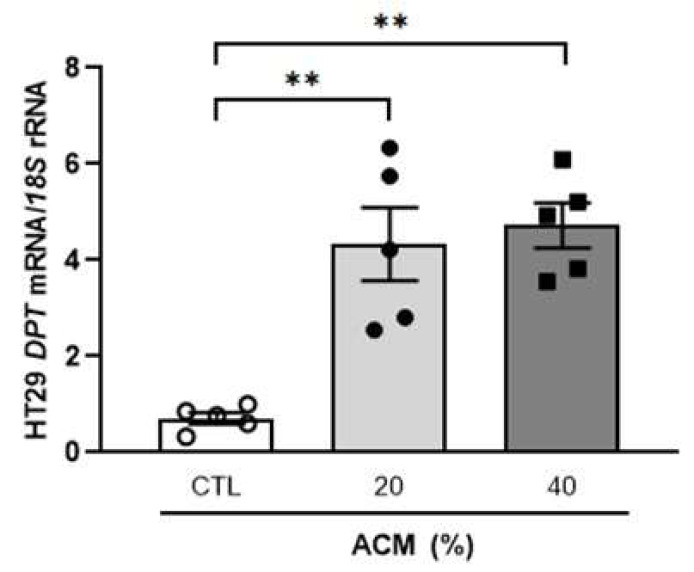
Effect of adipocyte-conditioned medium (ACM) from subjects with obesity on the gene expression levels of DPT in HT-29 cells. The bar graph represents the mean ± SEM (n = 5 per group). Differences were analysed by one-way ANOVA followed by Dunnett’s *post hoc* tests. ** *p* < 0.01.

To study the possible effect that the molecules released by visceral adipocytes from patients with OB may have on colon cancer cells, we analysed the expression of DPT in HT-29 cells treated with the adipocyte conditioned medium (ACM). Interestingly, we observed a strong (*p* < 0.01) increase in DPT mRNA levels in HT-29 cells treated with the ACM from volunteers with OB compared with the colon cancer cells incubated with the control media (Figure 4). Increased (*p* < 0.05) mRNA levels of DPT after ACM treatment were confirmed in Caco-2 cells (Supplemental Figure S2).

## 3. Discussion

Obesity, a current global pandemic, is considered a preventable risk factor for CC development [27,28,29,30]; moreover, a worse cancer prognosis has been described among patients with fat excess [27,31]. Increasing evidence reveals that obesity prompts the development of CC through the pathophysiological effects of VAT that favours tumourigenesis, tumour growth and metastasis. Especially, dysfunctional VAT in patients with obesity results in a systemic dysregulation of adipokines promoting a chronic inflammation state and changes in ECM remodelling that contributes to cancer development [32,33,34,35]. Since the excess deposition of ECM components, together with fibrosis, contribute to the development of CC, the study of the ECM microenvironment is considered of key importance [6,35]. In the present study, we explored the impact of DPT, a small protein of the ECM, in obesity-associated CC and we have found that (i) circulating levels of DPT and its gene expression levels in the colon are decreased in patients with CC and, oppositely, a tendency towards increased mRNA levels in VAT was found, (ii) the inflammation-related factors LPS and TNF-α upregulated DPT expression while IL-4 downregulated its expression in HT-29 cells, (iii) DPT influences the expression of critical ECM remodelling-related factors in colon cancer cells and iv) visceral adipocyte-derived factors from patients with OB increased the mRNA levels of DPT in HT-29 cells.

The decreased circulating concentrations of DPT and its expression levels in the colon found in patients with CC are in line with previous findings showing reduced *DPT* expression in several malignancies including hepatocellular carcinoma as well as oral, breast, ovarian, colon and papillary thyroid cancers [22,23,24,25,26,36]. In this regard, a tumour suppressor role for DPT has been proposed, mainly based on its role in increasing the biological activity of TGF-β [20,22]. TGF-β and its signalling effectors influence CC behaviour showing dual roles depending on the stage of tumour development [37,38]. Thus, TGF-β is considered both a tumour suppressor gene by inhibiting early stages of colon tumourigenesis and a significant stimulator of tumour progression, invasion and metastasis in more advanced stages [37]. Since the colon is anatomically located close to abdominal VAT, the study of the influence of dysfunctional visceral adipocytes during OB on cancer cells is of key importance to better understand the underlying mechanisms that link OB and CC. We found a tendency towards upregulation of *DPT* mRNA expression in the VAT from patients with CC, suggesting that in this tumour-surrounding tissue, DPT may promote CC development by favouring a pro-inflammatory microenvironment. In this sense, our group previously described that increased DPT levels in the VAT from individuals with OB promote fibrosis and inflammation [16]. In light of these findings, DPT may exhibit a different behaviour depending on the tissue in which it is expressed suggesting a tumour suppressor role in the colon, the tissue where cancer develops, and conversely, a promoter role of matrix remodelling and fibrosis in VAT.

We observed that the inflammation-related molecules LPS and TNF-α increased DPT expression while the anti-inflammatory cytokine IL-4 downregulated DPT expression in HT-29 cells. Since inflammation predisposes to the development of cancer and promotes all stages of tumourigenesis [39] and DPT was previously described as a tumour suppressor molecule, our findings suggest a compensatory mechanism whereby DPT expression may increase in the context of strong inflammation, trying to avoid or minimize cell damage exhibiting its tissue-repairing functions; however, whether DPT is an inflammation driver or a responder still remains unknown. A similar response was observed after an inflammatory insult in zebrafish and mice where DPT levels were increased after myeloablative radiation [40]. Furthermore, plasma levels of DPT also increase after chemotherapy treatments [40]. Importantly, TGF-β treatment induced an increase in the mRNA levels of DPT. A similar response was found in a human stellate LX-2 cell line and in cultured fibroblasts where DPT expression was inducible by TGF-β [18,41]. In line with our results, IL-4 has been involved in the persistent downregulation of DPT expression in skin fibroblasts in patients with systemic sclerosis, a fibrotic disorder [41]. Further research will help us to better understand the crosstalk between these important molecules.

The remodelling of the ECM is a dynamic event with a repairing role being proposed for DPT. Collagen is the main component of the tumour microenvironment and its content and distribution are modified to further participate in cancer fibrosis by coordinating the expression of different transcription factors, signal transduction pathways, and receptors [42]. Interestingly, we observed increased expression of *COL1A1* and *COL5A3* after the treatment with DPT at the highest concentration. Reportedly, *Dpt*-deficient mice display decreased collagen accumulation in the skin probably due to the loose packing of collagen fibrils and their irregular morphology [17]. During different phases of fibrosis development in tumour cells, soluble mediators including DPT may promote an irregular accumulation and composition of collagen, affecting the development of cancer. DPT is known to interact with decorin to enhance collagen fibrillogenesis [43]. We found reduced levels of *DCN* after DPT treatment. While DPT triggers a small diameter collagen assembly [44], decorin notably delays fibril formation [45], suggesting the role of these two molecules in the regulation of the assembly and turnover of collagen in the ECM greatly influencing cell behaviour in the tumour microenvironment [41]. Elegantly, Mao et al. described that the intestine of *Dcn*-deficient mice acquires a pro-tumourigenic phenotype by the increase of epithelial-mesenchymal transition markers that favours CC metastasis [46]. Previous studies showed that DPT enhances the expression and activity of TGF-β in other cell types [20,47], but we did not find changes in *TGFB* expression after treatment with DPT. Osteopontin (*SPP1*) is overexpressed in CC being associated with a poor prognosis linked to invasion and metastasis [48,49,50]. The decreased mRNA levels of *SPP1* after DPT treatment may suggest a role of DPT in reducing tumour progression [51]. VEGF proteins are involved in blood vessel formation in physiological and pathological events including wound healing, inflammatory diseases and cancer [52]. When tissues are damaged, VEGF-A is upregulated to form new capillaries ensuring immune cells, nutrients and oxygen supply to the injured area. *VEGFA* expression was increased after the stimulation with DPT suggesting that DPT may favour tissue repair by facilitating the angiogenesis process. Importantly, changes in gene expression levels were observed after the treatment with the highest concentration suggesting a threshold in DPT levels that would explain the dual behaviour that this molecule exhibits; however, not only the concentrations of this molecule may determine its biological effect, but also the availability and expression levels of the receptors to which DPT binds; these interactions can result in the activation of certain intracellular pathways or in the suppression of the functions of the receptor, as in the case of binding to the receptor VLA-4 [40]. Based on these data, we presume that DPT influences ECM remodelling in HT-29 cells by modulating the expression of certain types of collagens and molecules that are dysregulated in inflammation, obesity or cancer.

Specific adipokines secreted by dysfunctional adipocytes in OB have been shown to exert modifications of tumour cell behaviour probably by the induction of inflammation and ECM remodelling [53,54,55,56]. In our study, the increased gene expression levels of DPT in HT-29 cells after the stimulus with the ACM from patients with OB reflect the crosstalk between dysfunctional adipocytes and cancer cells.

The study has a few limitations. Further studies in larger cohorts to improve our understanding of the role of DPT in obesity-associated CC are needed and more studies on the dissection of signalling mechanisms of DPT-mediated angiogenesis might unravel novel targets for tissue regeneration and cancer. The results may be also verified in animal models since HT-29 cells do not represent a real tumour as they are a cultured cell line isolated from an adenocarcinoma. Thus, the cell variety within a tumour and/or different types of CC was not considered in this study. In this sense, the treatment of different tumoural cell lines including HCT116 +/− p53 due to the importance of p53 mutations and the microsatellite instability in CC would provide important and additional information about the role of DPT in the carcinogenesis process.

In light of these findings, the decreased circulating and expression levels of DPT in the colon together with the tendency towards increased levels of visceral AT in patients with CC suggest a possible role of DPT in the OB-associated CC. DPT could have a different behaviour being modulated in a time- or tissue-specific manner. In early tumour stages DPT could act as a tumour suppressor, while in other situations, this molecule could promote metastasis by favouring cell migration and fibrosis in the tumour microenvironment.

## 4. Materials and Methods

### 4.1. Patient Selection

**Figure 5:**
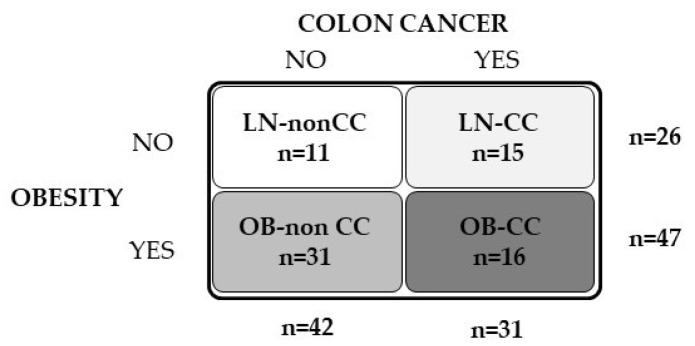
Schematic diagram showing the experimental groups and the sample size of volunteers included in the study.

In order to analyze the effect of obesity and CC on DPT concentrations, 73 subjects (26 LN and 47 with OB) were recruited from healthy volunteers and patients attending the Departments of Endocrinology and Nutrition and Surgery at the Clínica Universidad de Navarra (Figure 5). Patients were classified according to body mass index (BMI) (LN: BMI < 25 kg/m^2^ and OB: BMI > 30 kg/m^2^). BMI was calculated as weight in kilograms divided by the square of height in meters and body fat percentage (BF) was estimated using the Clínica Universidad de Navarra-Body Adiposity Estimator (CUN-BAE) [57]. Waist circumference was measured at the midpoint between the iliac crest and the rib cage on the midaxillary line. Subjects were further subclassified according to the established diagnostic protocol for CC [42 without CC (non-CC) and 31 with CC (pathological characteristics are shown in Supplemental Table S1)]. The control volunteers were healthy, were not receiving any pharmacological treatment and had no signs or clinical symptoms of cancer, liver alteration or T2D. VAT samples were collected from patients undergoing Nissen fundoplication (for LN volunteers), Roux-en-Y gastric bypass (for severe obesity) and curative resection for primary colon carcinoma (for CC treatment) [non-CC: n = 11; CC: n = 10] at the Clínica Universidad de Navarra. A cohort of human colon RNA samples (5 from normal colon tissue and 7 from CC tissue) were obtained from OriGene (Rockville, MD, USA). All reported investigations were carried out in accordance with the principles of the Declaration of Helsinki, approved by the Hospital’s Ethical Committee (protocol approval number: 2018.094) and informed consent from all volunteers was obtained.

### 4.2. Analytical Measurements

Blood samples were obtained after more than 8-h of fasting. Concentrations of glucose were measured by an automated analyser (Hitachi Modular P800, Roche, Basel, Switzerland) and triglycerides and free fatty acids levels were analysed by commercially available kits (Infinity^TM^, Thermo Electron Corporation, Melbourne, Australia) as previously described [58]. The high sensitivity CRP, fibrinogen and CEA concentrations were measured as previously described [59]. Serum concentrations of DPT were determined by using a commercially available ELISA kit according to the manufacturer’s instructions (Cusabio, Wuhan, China). The intra- and interassay coefficients of variation were 8% and 10%, respectively.

### 4.3. RNA Isolation and Real-Time PCR

RNA isolation was performed as previously reported [59]. The transcript levels for *COL1A1*, *COL5A3*, collagen type 6 α3 chain (*COL6A3*), *DCN*, DPT, *IL1B*, *IL8*, *IL18*, kruppel-like factor 4 (*KLF4*), matrix metalloproteinase-9 (*MMP9*), *SPP1*, *TGFB* and *VEGFA* were quantified by Real-Time PCR (7300 Real-Time PCR System, Applied Biosystem, Foster City, CA, USA) as previously described [59]. Primers and probes were designed using the software Primer Express 2.0 (Applied Biosystems, Foster City, CA, USA) and obtained from Genosys (Merck, Darmstadt, Germany). To avoid genomic DNA amplification, primers or TaqMan^®^ probes were designed to encompass fragments of the areas from the extremes of two exons (Supplemental Table S2).

The TaqMan^®^ Universal PCR Master Mix (Applied Biosystems) was used for cDNA amplification and the primer and probe concentrations for gene amplification were 300 nM and 200 nM, respectively. The endogenous control gene was the *18S* rRNA (Applied Biosystems) and the ΔΔCt formula was used for relative quantification. Relative gene expression levels were expressed as fold expression over the calibrator sample (average of individuals without CC or unstimulated HT-29 cells). All samples were run in triplicate and negative controls were included in all reactions.

### 4.4. Cell Cultures

Human stromovascular fraction cells (SVFC) were isolated from VAT in subjects with obesity. SVFC were cultured and differentiated to mature adipocytes as previously described [60] and the ACM was obtained from these cultures, centrifuged and diluted to 20% and 40%.

Two colorectal adenocarcinoma cell lines, HT-29 (HTB-38TM) and Caco-2 (HTB-37TM) were obtained from the ATCC^®^ (Middlesex, UK) and cultured following the manufacturer’s instructions. The Caco-2 cell line was a generous gift of Dr. Amaya Azqueta from the University of Navarra. Briefly, cells were seeded at 3 × 10^5^ cells/well and grown in McCoy’s 5A medium with L-glutamine (Merck, Darmstadt, Germany) supplemented with 10% fetal bovine serum and antibiotic–antimycotic at 37 °C for 24 h. HT-29 cells were serum-starved for 2 h and then treated with ACM (20% and 40%), DPT (1, 10 and 100 ng/mL) (R&D Systems, Minneapolis, MN, USA), IL-4 (1, 10 and 100 ng/mL) (R&D Systems, Minneapolis, MN, USA), IL-13 (1, 10, and 100 ng/mL) (R&D Systems), LPS (10, 100 and 1000 ng/mL) (R&D Systems), TGF-β (1, 10 and 100 ng/mL) (R&D Systems) and TNF-α (1, 10 and 100 ng/mL) (Sigma) for 24 h. Caco-2 cells were also serum-starved for 2 h and then stimulated with DPT (1, 10 and 100 ng/mL) and ACM (20 and 40%).

### 4.5. Statistical Analysis

Data are shown as mean ± standard error of the mean (SEM). Gene expression levels and CRP concentrations were logarithmically transformed due to their non-normal distribution. Differences between groups were assessed by two-way ANOVA, one-way ANOVA followed by Tukey’s or Dunnett’s *post hoc* tests and two-tailed unpaired Student’s *t*-tests as appropriate. Differences between groups adjusted for age were analysed by analysis of covariance (ANCOVA). The calculations were performed using the SPSS version 23 (SPSS, Chicago, IL, USA) and GraphPad 8.0 (San Diego, CA, USA). A *p*-value of <0.05 was considered statistically significant.

## Figures and Tables

**Table 1:** Characteristics of the volunteers included in the study.

	Lean		OB				
	non-CC	CC	non-CC	CC	*p* OB	*p* CC	*p* OBxCC
n (male, female)	11 (7, 4)	15 (8, 7)	31 (19, 12)	16 (9, 7)			
Age (years)	53 ± 3	63 ± 3	55 ± 1	62 ± 3	0.518	<0.001	0.925
Body weight (kg)	65.4 ± 1.9	63.4 ± 1.8	84.9 ± 2.1	81.1 ± 2.9	<0.001	0.303	0.753
Body mass index (kg/m^2^)	23.1 ± 0.2	22.5 ± 0.4	36.7 ± 0.7	34.2 ± 0.8	<0.001	0.065	0.772
Estimated body fat (%)	27.8 ± 2.2	29.1 ± 1.4	36.7 ± 1.2	34.2 ± 1.8	<0.001	0.268	0.369
Waist circumference (cm)	83 ± 2	80 ± 2	100 ± 2	115 ± 7	<0.001	0.270	0.228
Fasting glucose (mg/dL)	103 ± 5	142 ± 12	113 ± 5	127 ± 11	0.775	0.020	0.221
Free fatty acids (mg/dL)	11.7 ± 1.5	26.5 ± 2.4	15.2 ±1.2	22.6 ± 2.1	0.893	<0.001	0.086
Triglycerides (mg/dL)	93 ± 15	116 ± 11	118 ± 13	151 ± 28	0.079	0.398	0.324
CRP (mg/L)	0.90 ± 0.07	1.39 ± 0.82	1.63 ± 0.09	9.64 ± 1.49 ***	<0.001	<0.001	<0.001
Fibrinogen (mg/dL)	330 ± 18	273 ± 26	303 ± 20	447 ± 48 ^‡^	0.203	0.398	0.017
CEA (ng/mL)	1.58 ± 0.32	2.55 ± 0.44	1.68 ± 0.28	8.41 ± 2.60	0.267	0.021	0.401

Data are mean ± SEM. CC, colon cancer, CEA, *carcinoembryonic antigen*; CRP, C-reactive protein; OB, obesity. Statistical differences were analyzed by two-way ANCOVA and one-way ANCOVA followed by Tukey’s *post hoc* tests as appropriate. *** *p* < 0.001 vs. LN non-CC, LN-CC and OB non-CC. ^‡^ *p* < 0.05 vs. OB non-CC.

## Data Availability

The data that support the findings of this study are available from the corresponding author upon reasonable request.

## Supplementary Materials

The following supporting information can be downloaded at: https://www.mdpi.com/article/10.3390/ijms23169222/s1.
