# Supplementary material for: Dermatopontin Influences the Development of Obesity-Associated Colon Cancer by Changes in the Expression of Extracellular Matrix Proteins
Source: Int J Mol Sci. 2022 Aug 17;23(16):9222. doi: 10.3390/ijms23169222 (PMC9408942; doi:10.3390/ijms23169222)
Supplement: Supplementary file 1 [file ijms-23-09222-s001.zip › ijms-1747408-SI.pdf]

**Supplemental Table S1. Clinicopathological characteristics of patients with CC.**

| | |
|---|---|
| **Gender, n** | |
| Male | 17 |
| Female | 14 |
| **Location of primary lesion, n** | |
| Transverse colon | 4 |
| Right hemicolon | 12 |
| Left hemicolon | 13 |
| Missing | 2 |
| **TNM stage, n** | |
| I | 4 |
| II | 6 |
| III | 15 |
| IV | 4 |
| Missing | 2 |
| **Differentiation, n** | |
| Well | 3 |
| Moderately | 23 |
| Poorly and undifferentiated | 3 |
| Missing | 2 |
| **Tumor size, n** | |
| < 5 cm | 16 |
| > 5 cm | 7 |
| Missing | 8 |
| **Lymph node status, n** | |
| Positive | 9 |
| Negative | 22 |

**Supplemental Table S2. Sequences of primers and probes.**

| Gene (GenBank accession) | Oligonucleotide sequence (5′-3′) |
|---|---|
| *COL1A1* (NM_000088.3) | |
| Forward | CTCCCGGGCCTCAAGGTAT |
| Reverse | TTGCTCCAGAGGGACCTTGTT |
| TaqMan® Probe | FAM-TCTTCCTGGCCCCTCTGGTGAACCT-TAMRA |
| *COL5A3* (NM_015719.4) | |
| Forward | GAACAAGGAAATTTGGACCTCAAG |
| Reverse | GATTTGGAGCTGGAGTCTCTGTCT |
| TaqMan® Probe | FAM-TCCTGACTCCGCAGAGAACCAGACCTC-TAMRA |
| *COL6A3* (NM_004369.3) | |
| Forward | GACGGAGATCTGGCTGATTTACA |
| Reverse | AGATGCATTAGCCGCTCCAA |
| TaqMan® Probe | FAM-AGAACCTCCGCCAAGAAGGAGTCCGT-TAMRA |
| *DCN* (NM_001920.5) | |
| Forward | AGAAGCTCTCCTACATCCGCATT |
| Reverse | CTGCATCAACTCTGCTGATTTGT |
| TaqMan® Probe | FAM-TTCCTCAAGGTCTTCCTCCTTCCCTTACG-TAMRA |
| *DPT* (NM_001937.5) | |
| Forward | GGCAGTTTTACTGTTGTCGCTACA |
| Reverse | CATGTCCATTTCCTCACCATAGTG |
| TaqMan® Probe | FAM-TGCCCATATTCCTGCTGGCTAACAACAG-TAMRA |
| *IL1B* (NM_000576) | |
| Forward | CAGTGGCAATGAGGATGACTTG |
| Reverse | GTAGTGGTGGTCGGAGATTCGTA |
| TaqMan® Probe | FAM-TGGCCCTAAACAGATGAAGTGCTCCTTCC-TAMRA |
| *IL8* (NM_000584.3) | |
| Forward | ACCTTTCCACCCCAAATTTATCA |
| Reverse | TTCTCAGCCCTCTTCAAAAACTTC |
| TaqMan® Probe | FAM-CCACACTGCGCCAACACAGAAATTATTGTA-TAMRA |
| *IL18* (NM_001562) | |
| Forward | CCAAGGAAATCGGCCTCTATT |
| Reverse | CCTCTAGGCTGGCTATCTTTATACATACT |
| TaqMan® Probe | FAM-TTCTGACTGTAGAGATAATGCACCCCGGAC-TAMRA |
| *KLF4* (NM_001314052.1) | |
| Forward | ACCTACACAAAGAGTTCCCATCTCA |
| Reverse | GTTTACGGTAGTGCCTGGTCAGTT |
| TaqMan® Probe | FAM-CCTGCGAACCCACACAGGTGAGAAA-TAMRA |
| *MMP9* (NM_004994) | |
| Forward | GCCCGGACCAAGGATACAGT |
| Reverse | CCCCTCAGTGAAGCGGTACA |
| TaqMan® Probe | FAM-ACGCGCTGGGCTTAGATCATTCCTCA-TAMRA |
| *SPP1* (NM_000582) | |
| Forward | CATCCAGTACCCTGATGCTACAGA |

| | |
|---|---|
| Reverse | GGCCTTGTATGCACCATTCAA |
| TaqMan® Probe | FAM-ACATCACCTCACACATGGAAAGCGAGGA-TAMRA |
| *TGFB* (NM_000660) | |
| Forward | GCCCAGCATCTGCAAAGC |
| Reverse | TCCTTGCGGAAGTCAATGTACA |
| TaqMan® Probe | FAM-CACCAACTATTGCTTCAGCTCCACGGA-TAMRA |
| *VEGFA* (NM_001025250) | |
| Forward | CAGCACAACAAATGTGAATGCA |
| Reverse | ACACGTCTGCGGATCTTGTACA |
| TaqMan® Probe | FAM-AATCCCTGTGGGCCTTGCTCAGAGC-TAMRA |

*COL*, collagen; *DCN*, decorin; *DPT*, dermatopontin; *IL*, interleukin; *KLF4*, Kruppel-like factor 4; *MMP9*, matrix metalloproteinase 9; *SPP1*, osteopontin; *TGFB*, transforming growth factor-β; *VEGFA*, vascular endothelial growth factor A.

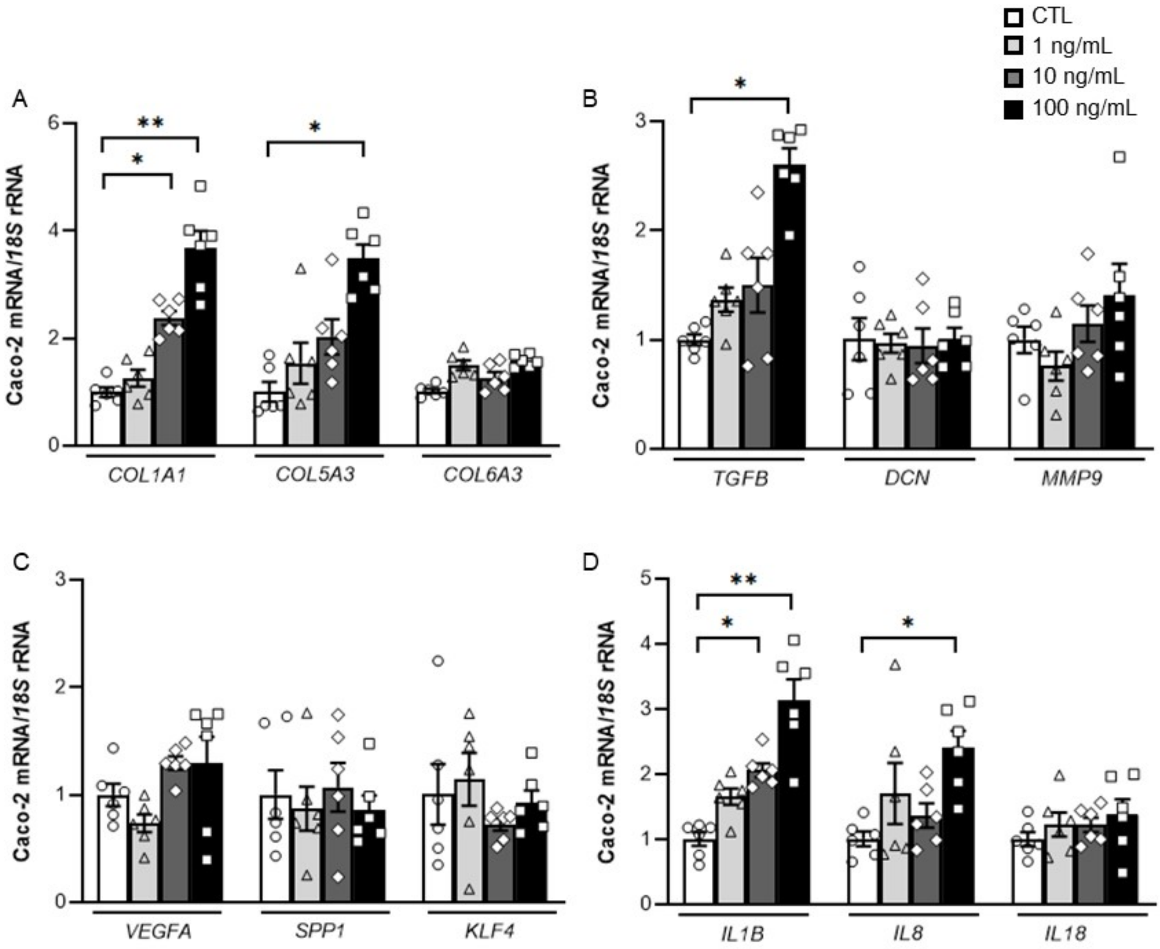


**Supplemental Figure S1.** Effect of DPT treatment on the expression levels of A) collagen (*COL*)-*1A1*, *COL5A3* and *COL6A3*; B) transforming growth factor-β (*TGFB*), decorin (*DCN*) and matrix metalloproteinase (*MMP*)-*9*; C) vascular endothelial growth factor A (*VEGFA*), osteopontin (*SPP1*) and Kruppel-like factor 4 (*KLF4*) and D) interleukin (*IL*)-*1B*, *IL8* and *IL18* in Caco-2 cells. Values are the mean ± SEM (n = 6 per group). Differences between groups were analysed by one-way ANOVA followed by Dunnett's *post hoc* tests. * $P<0.05$ and ** $P<0.01$.

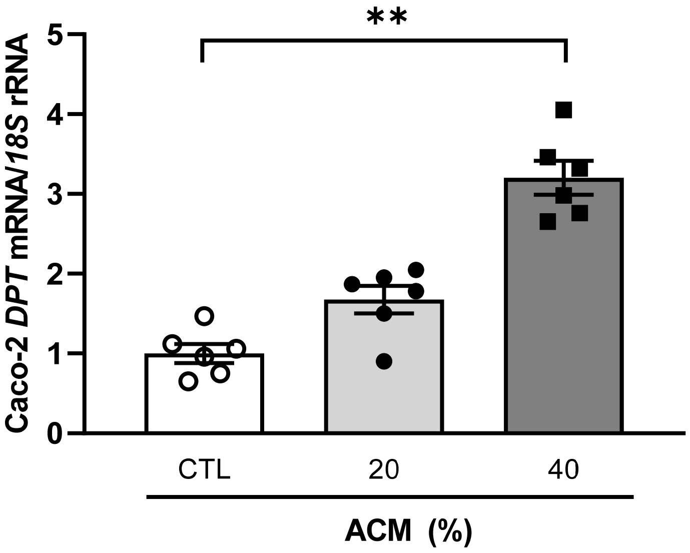


**Supplemental Figure S2**. Effect of adipocyte-conditioned media (ACM) on the gene expression levels of *DPT* in Caco-2 cells. Bar graph represents the mean ± SEM (n = 6 per group). Differences were analysed by one-way ANOVA followed by Dunnett's *post hoc* tests. ** *P*<0.01.
